# Efficacy and Safety of Omalizumab and Dupilumab in Pediatric Patients with Skin Diseases: An Observational Study

**DOI:** 10.3390/jpm15020064

**Published:** 2025-02-07

**Authors:** Francesca Galletta, Ludovica Rizzuti, Stefano Passanisi, Emanuela Rosa, Lucia Caminiti, Sara Manti

**Affiliations:** Pediatric Unit, Department of Human Pathology in Adult and Developmental Age “Gaetano Barresi”, University of Messina, Via Consolare Valeria 1, 98124 Messina, Italy; francygall.92@gmail.com (F.G.); ludovicarizzuti@gmail.com (L.R.); emanuelarosamed@gmail.com (E.R.); lucia.caminiti@polime.it (L.C.); sara.manti@unime.it (S.M.)

**Keywords:** biologics, chronic spontaneous urticaria, moderate-to-severe atopic dermatitis, efficacy, safety

## Abstract

**Background:** Chronic spontaneous urticaria (CSU) and moderate-to-severe atopic dermatitis (AD) are significant challenges in pediatric populations, negatively impacting quality of life (QoL). Biologic therapies, including omalizumab and dupilumab, showed considerable promise for patients unresponsive to conventional treatments. This study evaluated the real-life efficacy and safety of these biologics in pediatric CSU and AD patients. **Methods:** A retrospective, monocentric study was conducted enrolling pediatric patients (aged 6–18 years) followed at the “G. Martino” Hospital, University of Messina. This study included patients with CSU unresponsive to antihistamines and those with moderate-to-severe AD refractory to topical therapies. Disease severity and treatment efficacy were evaluated using the Urticaria Activity Score 7 (UAS7) for CSU, the Eczema Area and Severity Index (EASI) for AD, and QoL metrics, including the Dermatology Life Quality Index (DLQI) and numerical rating scales, for pruritus (p-NRS) and sleep (s-NRS), at baseline, 16 weeks, and 52 weeks. Safety was assessed through the monitoring of reported adverse events (AEs). **Results:** Omalizumab significantly reduced UAS7 scores by 71.9% at 16 weeks and 75.3% at 52 weeks (*p* < 0.001), with concurrent improvements in c-DLQI. Dupilumab reduced the EASI score by 75.3%, p-NRS by 40%, and s-NRS by 52.9% over 52 weeks, with c-DLQI improving by 72.6%. No severe AEs were observed; mild reactions included injection-site erythema and respiratory symptoms. **Conclusions:** Omalizumab and dupilumab demonstrated significant efficacy in reducing disease severity and improving QoL in pediatric patients with CSU and AD. Moreover, their safety profile underscores their potential as essential treatments for these conditions.

## 1. Introduction

Therapeutic management of many diseases has progressively shifted from a standardized to a personalized approach, focusing on the inhibition of specific molecular targets by monoclonal antibodies [1,2]. These biologics include antibodies targeting Th2-type inflammatory mediators and cytokines produced by pro-inflammatory cells of the innate immune system, characteristic of non-Th2-type inflammation [2]. Monoclonal antibodies have wide applicability in managing skin disorders such as chronic spontaneous urticaria (CSU) and moderate-to-severe atopic dermatitis (AD). Among these, omalizumab and dupilumab are the most widely used for children and adolescents with these conditions [2]. CSU is estimated to affect 1.4% of the pediatric population [3]. The prevalence of AD among children is approximately 20%, with substantial variations across countries [4].

Omalizumab, a humanized IgG1κ monoclonal antibody, binds to IgE, preventing its interaction with IgE receptors and reducing pro-inflammatory mediators, mast cell activation, and eosinophil infiltration [5]. It was approved by the Food and Drugs Administration (FDA) and European Medicine Agency (EMA) in 2014 for the treatment of CSU in youth ≥12 years who have shown inadequate response to antihistamines [6,7]. Additionally, the Italian Medicines Agency (AIFA) has also approved its use, allowing omalizumab to be prescribed and reimbursed under the same criteria [4,5]. Dupilumab, a fully human IgG4 monoclonal antibody directed against IL-4Rα, blocks IL-4 and IL-13 signaling pathways, thereby mitigating Th2-mediated inflammation [8]. Initially approved in 2019 for adolescents (aged 12–17 years) with moderate-to-severe AD, its indication was later expanded to include children aged 6–11 years in 2020 and infants as young as six months in 2023 [9]. In Italy, dupilumab is eligible for prescription and reimbursement in children and adolescents aged 6 to 17 years with severe atopic dermatitis requiring systemic therapy, provided they have an EASI score ≥ 24 or fulfill at least one of the following criteria: localization in visible and/or sensitive areas; pruritus assessment with an NRS score ≥ 7; quality of life assessment with a CDLQI score ≥ 10 [9]. Several randomized clinical trials (RCTs) and post-marketing studies have demonstrated the effectiveness and safety of these biologics in reducing disease severity, minimizing adverse events (AEs), and improving quality of life (QoL) over the medium and long term [2]. In recent years, the literature has increasingly emphasized studies conducted in real-life settings, as they provide a more accurate reflection of the effectiveness and safety of biologics in everyday clinical practice, complementing the findings from RCTs [2].

Our study aimed to evaluate the effectiveness and safety profiles of omalizumab and dupilumab in a real-life setting over a 52-week period in a pediatric population diagnosed with CSU unresponsive to antihistamines and moderate-to-severe AD.

## 2. Materials and Methods

An observational, ambispective, monocentric real-life study was conducted at the Pediatric Unit of the “G. Martino” Hospital, University of Messina, Italy. Data collection was planned to include a retrospective component starting in February 2020 and a prospective component extending up to June 2024. Children aged 6–18 years were consecutively enrolled based on the following criteria: a diagnosis of CSU that remained uncontrolled despite treatment with H1-antihistamines administered up to four times daily, with a Urticaria Activity Score 7 (UAS7) ≥ 16, or moderate-to-severe AD that was unresponsive to conventional topical or systemic therapies, including emollient creams, topical corticosteroids (TCSs), oral corticosteroids (OCSs), topical calcineurin inhibitors (TCIs), or ciclosporin, with an Eczema Area and Severity Index (EASI) ≥ 24 or in the presence of the above-mentioned criteria for dupilumab prescription according to AIFA [7,9,10]. Exclusion criteria included the concomitant use of systemic immunomodulating agents, previous use of omalizumab or dupilumab, a history of allergic or systemic reactions to biologics, other chronic conditions, or poor adherence to treatment. Informed consent was obtained from all participants and their parents. Efficacy was primarily assessed by evaluating disease activity using standardized, validated scoring systems: the UAS7 score for CSU and the EASI score for AD. QoL metrics were also assessed, including the Pruritus Numerical Rating Scale (p-NRS) and Sleep Numerical Rating Scale (s-NRS) for AD. Additionally, the Dermatology Life Quality Index (DLQI) was used for patients over 16 years of age, while the Children’s Dermatology Life Quality Index (c-DLQI), designed for children aged 4 to 16 years, was applied to evaluate QoL in both conditions. Anamnestic and clinical data—such as body mass index (BMI), comorbidities, and prior treatments—were also recorded. Safety was evaluated based on the incidence and characterization of AEs, categorized as mild, moderate, or severe according to criteria established by the EMA and FDA [4,7]. During follow-up visits, patients were asked about any AEs they experienced, with documentation following the terminology of the Medical Dictionary for Regulatory Activities (MedDRA®). Physicians recorded the severity, time of onset, need for hospitalization, and clinical outcomes of reported AEs. Assessments of efficacy and safety were conducted at baseline and at 16 and 52 weeks following the initiation of biologic therapies. This study was conducted according to Good Clinical Practice and in accordance with the Declaration of Helsinki [11]. Ethics committee approval was not required, as per the General Authorization to Process Personal Data for Scientific Research Purposes (Authorization No. 9/2014), which states that studies using identifier codes do not necessitate ethical approval.

### 2.1. Dosing

Omalizumab and dupilumab were administered via subcutaneous injection. For CSU, omalizumab was administered at a fixed dose of 300 mg every four weeks, indicated for children and adolescents aged 12 years and older who were eligible for systemic therapy [6]. Dupilumab dosing for patients with severe AD varied according to both age and body weight. For adolescents aged 12–17 years, patients weighing less than 60 kg received an initial dose of 400 mg, followed by 200 mg every two weeks. In contrast, those weighing over 60 kg were given an initial dose of 600 mg, followed by 300 mg every two weeks [9]. For patients younger than 12 years, the regimen consisted of an initial dose of 300 mg with subsequent doses of 300 mg every four weeks for those weighing less than 60 kg. Meanwhile, individuals weighing 60 kg or more received a starting dose of 600 mg, followed by 300 mg every two weeks [9].

### 2.2. Administered Questionnaire

#### 2.2.1. UAS7

The UAS7 is a widely used tool for assessing disease activity and response to treatment. It serves as an effective method for monitoring patients during follow-up visits [10]. The UAS7 score is calculated as the sum of daily symptom scores over seven consecutive days [12]. This scoring system classifies the severity of CSU as severe (28–42), moderate (16–27), mild (7–15), well-controlled (1–6), and absent (0). Additionally, it is instrumental in evaluating treatment efficacy [12].

#### 2.2.2. EASI Score

The EASI score is a commonly utilized tool for assessing the severity of AD. It evaluates erythema, excoriation, and lichenification based on the extent of skin involvement [13]. The EASI score ranges from 0, indicating no dermatitis or dermatitis in remission, to 72, representing severe dermatitis. However, the score does not account for subjective symptoms like sleep disruption or itch intensity, which require additional metrics for a comprehensive evaluation [14,15]. The EASI score is calculated by assessing the area of involvement and dividing the body into four regions: head and neck, upper limbs, trunk, and lower limbs. Each region is scored based on the percentage of skin affected, ranging from 0 (no involvement) to 6 (90–100% involvement). Further parameters evaluated were the severity of lesions (redness, swelling, scratching, skin thickening caused by chronic irritation). Each parameter is graded on a scale of 0 (no signs) to 3 (severe signs), with a combined maximum score of 12 across all parameters. The total EASI score is calculated by summing the area and severity scores [13].

#### 2.2.3. NRS

NRS is a standardized tool widely used to evaluate and quantify the severity of subjective symptoms, particularly itching (pruritus) and sleep disturbances, in individuals with AD. P-NRS allows patients to rate the severity of itching on a scale from 0 to 10, where 0 represents no itch and 10 represents the worst itch. Similarly, s-NRS ranges from 0 (“no sleep disturbance” or “sleep is not affected”) to 10 (“severe sleep disturbance” or “cannot sleep at all”) [14,15].

#### 2.2.4. c-DLQI

DLQI/c-DLQI is a 10-item questionnaire designed to assess the impact of dermatological conditions on a patient’s quality of life over the previous seven days. This tool is widely used to evaluate the quality of life of children and adolescents with skin diseases and to monitor treatment effectiveness over time. The questionnaire generates a total score ranging from 0 (indicating no impact on quality of life) to 30 (representing the highest possible impact) [16].

#### 2.2.5. Statistical Analysis

Numerical data were presented as mean ± standard deviation (SD), while categorical variables were reported as absolute frequencies and percentages. The normality of data distribution was verified using the Kolmogorov–Smirnov test, allowing for a parametric analytical approach. Group comparisons were conducted using ANOVA for three-group analyses, while pairwise comparisons were performed using Student’s t-test. The Bonferroni correction was applied, considering a significance threshold of *p* < 0.017. Statistical analyses were carried out using the Statistical Package for Social Sciences (SPSS), version 22.0.

## 3. Results

### 3.1. Study Population

A total of 30 pediatric patients were recruited, the majority of whom were female, accounting for 63.4% of the population, with a mean age of 14.7 years (±2.1 SD). The participants were divided into two distinct groups based on their diagnoses and treatment: 13 patients (43.3%) with severe CSU were treated with omalizumab, while the remaining 17 patients (56.7%) with moderate-to-severe AD received dupilumab. All patients completed the 52-week study period. More than half of the patients reported allergic rhinitis (56.6%), while nearly half experienced allergic conjunctivitis (46.6%). Asthma was present in over a third of the cohort (36.6%), and a smaller proportion (6.6%) reported food allergies. In addition to these atopic conditions, other comorbidities were observed. Obesity was the most common, affecting 26.7% of the participants, followed by Hashimoto’s thyroiditis (20%). Less frequent comorbidities included neurological disorders (6.7%) and Caroli’s disease (3%). The demographic and clinical characteristics of the study population are detailed in Table 1.

### 3.2. Omalizumab in CSU

**Figure 1:**
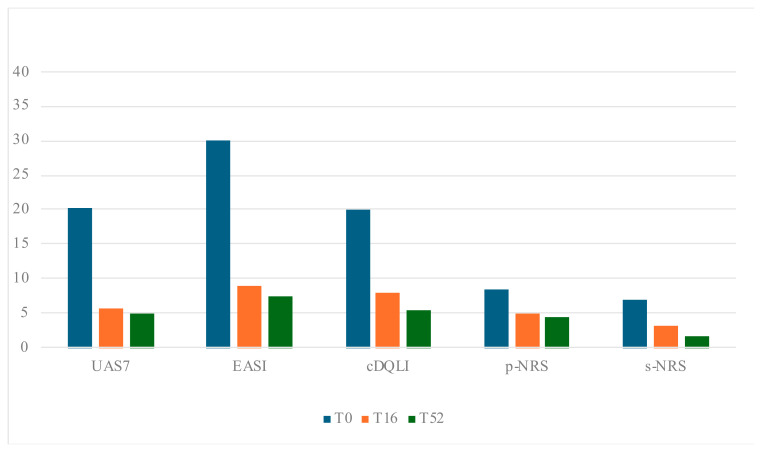
Changes in clinical scores across the study period; *p*-value < 0.001.

Among the 13 patients with CSU, 69.2% were female, and the mean age was 14.8 years (±1.55 SD). A significant reduction in the UAS7 score was observed, with a 71.9% decrease at 16 weeks (*p* < 0.001). This improvement remained consistent at 52 weeks, with a 75.3% reduction in the UAS7 score (*p* < 0.001). Additionally, patients experienced significant improvements in quality of life, as demonstrated by a substantial reduction in DLQI/c-DLQI scores at both 16 and 52 weeks (*p* < 0.001) (Table 2; Figure 1). No dose adjustments were required across the study period.

### 3.3. Dupilumab in AD

Among the 17 patients with moderate-to-severe AD, 58.8% were female, with a mean age of 14.6 years (±2.49 SD). Treatment with dupilumab resulted in a reduction in the mean EASI score by 70.3% at 16 weeks and 75.3% at 52 weeks compared to baseline. Furthermore, a significant decrease was observed in subjective symptoms, with the p-NRS score reduced by 40% and the s-NRS score by 52.9% (*p* < 0.001). DLQI/c-DLQI score demonstrated a significant decrease from the baseline value to the end of treatment (*p* < 0.001), with a mean percentage reduction from baseline to 52w of 72.6%. (Table 3; Figure 1). No dose adjustments occurred during the whole study period.

### 3.4. Safety

Patients receiving omalizumab for CSU did not experience any AEs. Among those treated with dupilumab, one patient (3.3%) reported erythema at the injection site, while two (6.7%) experienced upper respiratory tract symptoms, which were likely unrelated to the treatment. No serious AEs were observed during the follow-up period for either medication.

## 4. Discussion

### 4.1. Efficacy and Safety of Omalizumab

Our study confirmed the effectiveness of omalizumab in children in a real-world setting, demonstrated by a significant reduction in disease activity (UAS7) and improved quality of life (DLQI/c-DLQI) over the observational period. UAS7 scores showed a 75.3% reduction from baseline to 52 weeks, and a notable decrease in exacerbations. A systematic review of ten RCTs involving 1620 children and adults treated with omalizumab for 16–40 weeks further supports these outcomes [17]. At a dosage of 300 mg, omalizumab demonstrated statistically significant improvements in UAS7 scores (MD −11.05; 95% CI −12.87 to −9.24) and quality of life, as measured by DLQI scores (MD −4.03; 95% CI −5.56 to −2.5), with high-certainty evidence [17]. Additionally, a recent cumulative meta-analysis underscored the reliability of omalizumab’s efficacy over time, showing consistent outcomes across varying doses and sample sizes, with newer studies providing enhanced precision [16]. Real-world studies have consistently supported these findings [18,19,20,21,22]. For instance, a meta-analysis of 15 real-world studies, including 294 patients with CSU, reported a significant reduction in UAS7 scores (mean decrease of 25.6 points; 95% CI −28.2 to −23.0; *p* < 0.001) [19]. In real-life settings, improvements in DLQI/c-DLQI, and consequently in quality of life, were noteworthy [21]. A randomized study involving both adults and adolescents evaluated DLQI/cDLQI worsening (≥3 points) after treatment discontinuation [23]. Results indicated that patients in the placebo group were significantly more likely to experience a decline in DLQI compared to those in the omalizumab group (RR 3.34; 95% CI 2.07 to 5.40) [23]. Regarding the safety profile, no AEs were reported in our study. Findings from RCT and real-life studies consistently demonstrate that omalizumab is well tolerated, with a low frequency and severity of AEs in both short- and long-term use [24,25]. The most commonly reported AEs include fatigue, arthralgia, headache, and nausea [24,25]. A prospective real-world study conducted by our group, involving pediatric patients with asthma and/or CSU, reported no cases of anaphylaxis or severe AEs over a 4-year treatment period [26]. Similarly, a recent real-life retrospective study by Calzari et al., which included 296 patients with severe CSU, confirmed the sustained safety and effectiveness of omalizumab for managing CSU across an 8-year follow-up period [27].

### 4.2. Efficacy and Safety of Dupilumab

Our study confirmed the effectiveness of dupilumab in a pediatric population with moderate-to-severe AD, as demonstrated by significant reductions in objective disease severity scores (EASI), subjective symptom scores (p-NRS, s-NRS), and improvements in quality of life (DLQI/c-DLQI) during the observational period. Improvements across all measures were statistically significant between baseline and week 52. EASI scores decreased by approximately 75%, with most patients achieving complete or near-complete resolution of affected skin areas, and no progression of lichenification processes was observed. These results align closely with findings in the literature. Blauvelt et al. [28] supported the long-term efficacy of dupilumab in a study involving 294 adolescents aged ≥12 to 18 years, with 42.7% of patients experiencing a reduction in EASI scores at week 52. Similarly, Paller et al. [29], in an RCT involving children aged 6–12 years, showed significant improvements in EASI scores among patients treated with dupilumab compared to those receiving placebo. Real-world studies further supported these findings [30,31]. Patruno et al. [32] analyzed data from 36 Italian dermatological and pediatric centers involving children aged 6–11 years treated with dupilumab. Among the 96 children enrolled, 91 (94.8%) completed the 52-week treatment, with significant improvements in EASI, p-NRS, s-NRS, and c-DLQI scores observed at weeks 16, 24, and 52 [32]. Similarly, Hosseini-Hashrafi et al. [31], in a retrospective study, reported a median 94% decrease in EASI scores (IQR 82–100) that remained consistently low over a follow-up period of 10–52 months. Early therapeutic benefits were also noted, with statistically significant improvements in EASI, p-NRS, s-NRS, and quality of life parameters observed as early as 16 weeks [31]. Napolitano et al. [33] further reported substantial mean reductions in i-NRS, s-NRS, and c-DLQI scores (68.39%, 70.22%, and 79.03%, respectively) within just two weeks of treatment, with sustained benefits in subsequent weeks. Our study also demonstrated a favorable safety profile for dupilumab. Only one patient experienced mild erythema at the injection site, while two reported upper respiratory tract symptoms, which were likely unrelated to the medication. These results differ from the literature [34,35,36]. For instance, several RCTs found a higher incidence of conjunctivitis and injection-site reactions in dupilumab-treated patients compared to those receiving placebo [34,35,36]. Parmar et al. [37], in a long-term real-world study involving 128 pediatric and adult patients treated for an average duration of 14.90 ± 10.39 months, identified frequently reported AEs, including dermatitis affecting the head and neck (19.5%), conjunctivitis (15.6%), erythema, pruritus, skin peeling (10.9%), and ocular dryness (7.8%). Similarly, Patruno et al. [32] documented AEs in 14.8% of cases, with conjunctivitis (8.3%) and injection-site reactions (6.25%) being the most prevalent. Notably, no severe AEs were reported, and no children were forced to discontinue biological therapy [32]. A significant milestone in dupilumab’s history was its recent approval for use in infants as young as six months, where it has also proven to be effective and safe. The LIBERTY AD PRE-SCHOOL study, a randomized, double-blind, phase III trial, confirmed dupilumab’s efficacy and safety for children aged 6 months to 6 years with moderate-to-severe AD [35]. The study found that a significantly higher proportion of patients in the dupilumab group achieved EASI improvements compared to the placebo group (53% vs. 11%; *p* < 0.0001) [38]. Moreover, the incidence of skin infections and AD exacerbations was lower with dupilumab. However, a higher incidence of conjunctivitis was noted in the dupilumab group (5% vs. 0%) [38].

### 4.3. Strength and Limitations

One of the main limitations of this study is the small sample size, which inevitably reduces the statistical power of the study and affects the generalizability of the results. Additionally, the study population is composed of two distinct groups, each receiving biologics for different conditions. This distinction means that direct comparisons between omalizumab and dupilumab are not possible, as the two treatments target different immunological pathways. Despite their differences, both biologics are increasingly used in pediatric skin diseases. This study, therefore, provides a broader real-world perspective on the role of biologics in pediatric skin diseases rather than focusing on a single disorder. Moreover, the use of validated outcome measures ensures a systematic and standardized assessment of disease severity and treatment response.

## 5. Conclusions

In conclusion, omalizumab and dupilumab have demonstrated to be effective and safe treatments in managing severe skin conditions in children and adolescents. Their introduction represents a significant advancement in pediatric dermatology, offering meaningful improvements in disease outcomes and quality of life. Our results, in line with the existing literature, also underscores the critical importance of real-world evidence in evaluating biologic therapies. Real-life studies provide valuable insights that complement controlled trials by reflecting treatment effectiveness and safety across different patient populations and clinical settings. Such evidence is crucial for optimizing biologic use and tailoring treatment strategies to the specific needs of individual patients. Future research should prioritize real-world studies to expand the knowledge of long-term outcomes and the factors influencing treatment responses. This approach will support the development of more personalized and effective care strategies, ultimately improving patient outcomes and advancing the field of pediatric dermatology.

## Figures and Tables

**Table 1:** Demographic and clinical characteristics of study population.

Characteristics	CSU	AD	Overall
Number, no. (%)	13 (43.3)	17 (56.7)	30 (100)
Age (years), mean (SD)	14.8 (±1.55)	14.6 (±2.49)	14.7 (±2.1)
Female, no. (%)	9 (69.2)	10 (58.8)	19 (63.4)
BMI (kg/m^2^), mean (SD)	25.44 (±5.29)	22.65 (±3.92)	23.9 (±4.7)
**Other comorbidities, no. (%)**			
- Asthma	2/13 (15.3)	9/17 (52.9)	11/30 (36.6)
- Allergic rhinitis	5/13 (38.4)	12/17 (70.6)	17/30 (56.7)
- Allergic conjunctivitis	3/13 (23)	11/17 (64.7)	14/30 (46.6)
- Food allergy	0/13 (0)	2/17 (11.7)	2/30 (6.6)
- Obesity	6/13 (46.1)	2/17 (11.7)	8/30 (26.7)
- Hashimoto’s thyroiditis	2/13 (15.3)	1/17 (5.6)	3/30 (20)
- Neurological disorders	0/13 (0)	2/17 (11.7)	2/30 (6.7)
- Caroli’s disease	0/13 (0)	1/17 (5.6)	1/30 (3)
**Previous therapies, no (%)**			
- Oral corticosteroids	2/13 (15.4)	5/17 (29.4)	7/30 (23.3)
- Antihistamines	13/13 (100)	10/17 (58.8)	23/30 (76.6%)
- Topical corticosteroids	/	17/17 (100)	17/30 (56.7)
- Calcineurin inhibitors	/	5/17 (29.4)	5/30 (16.7)
- Ciclosporin	/	3/17 (17.6)	3/30 (10)

**BMI:** Body mass index.

**Table 2:** Statistical analysis of CSU group.

	T0	T16	p T0 vs. T16	T52	p T0 vs. T52
UAS7 mean (SD)	20.3 (±3.2)	5.7 (±3.6)	*p* < 0.001	5 (±2.5)	*p* < 0.001
DLQI/c-DLQI, mean (SD)	16.8 (±3.4)	4.4 (±3.5)	*p* < 0.001	3.30 (±1.9)	*p* < 0.001

**UAS7**: Urticaria Activity Score 7; **c-DLQI**: Children’s Dermatology Life Quality Index.

**Table 3:** Statistical analysis of moderate–severe AD group.

	T0	T16	p T0 vs. T16	T52	p T0 vs. T52
EASI, mean (SD)	30.0 (±14.9)	8.9 (±6.6)	*p* < 0.001	7.4 (± 5.3)	*p* < 0.001
p-NRS, mean (SD)	8.5 (±1.1)	5.1 (± 2.8)	*p* < 0.001	4.5 (±2.5)	*p* < 0.001
s-NRS, mean (SD)	7.0 (±2.7)	3.3 (±3.1)	*p* < 0.001	1.8 (±2.4)	*p* < 0.001
DLQI/c-DLQI, mean (SD)	20.1 (±6.3)	8.1 (±4.0)	*p* < 0.001	5.5 (±3.2)	*p* < 0.001

**EASI**: Eczema Area and Severity Index; **p-NRS:** pruritus Numerical Rating Scale; **s-NRS:** sleep Numerical Rating Scale; **DLQI/c-DLQI**: Dermatology Life Quality Index/Children’s Dermatology Life Quality Index.

## Data Availability

Data are contained within the article.
